# Therapeutic potential of extracellular vesicles derived from human mesenchymal stem cells in a model of progressive multiple sclerosis

**DOI:** 10.1371/journal.pone.0202590

**Published:** 2018-09-19

**Authors:** Fernando Laso-García, Jaime Ramos-Cejudo, Francisco Javier Carrillo-Salinas, Laura Otero-Ortega, Ana Feliú, MariCarmen Gómez-de Frutos, Miriam Mecha, Exuperio Díez-Tejedor, Carmen Guaza, María Gutiérrez-Fernández

**Affiliations:** 1 Neuroscience and Cerebrovascular Research Laboratory, Department of Neurology and Stroke Center, La Paz University Hospital, Neuroscience Area of IdiPAZ Health Research Institute, Autonomous University of Madrid, Madrid, Spain; 2 Functional and Systems Neurobiology Department, Neuroimmunology Group, Cajal Institute, Madrid, Spain; Fraunhofer Research Institution of Marine Biotechnology, GERMANY

## Abstract

Extracellular vesicles (EVs) have emerged as important mediators of intercellular communication and as possible therapeutic agents in inflammation-mediated demyelinating diseases, including multiple sclerosis (MS). In the present study, we investigated whether intravenously administered EVs derived from mesenchymal stem cells (MSCs) from human adipose tissue might mediate recovery in Theiler’s murine encephalomyelitis virus (TMEV)-induced demyelinating disease, a progressive model of MS. SJL/J mice were subjected to EV treatment once the disease was established. We found that intravenous EV administration improved motor deficits, reduced brain atrophy, increased cell proliferation in the subventricular zone and decreased inflammatory infiltrates in the spinal cord in mice infected with TMEV. EV treatment was also capable of modulating neuroinflammation, given glial fibrillary acidic protein and Iba-1 staining were reduced in the brain, whereas myelin protein expression was increased. Changes in the morphology of microglial cells in the spinal cord suggest that EVs also modulate the activation state of microglia. The clear reduction in plasma cytokine levels, mainly in the Th1 and Th17 phenotypes, in TMEV mice treated with EVs confirms the immunomodulatory ability of intravenous EVs. According to our results, EV administration attenuates motor deficits through immunomodulatory actions, diminishing brain atrophy and promoting remyelination. Further studies are necessary to establish EV delivery as a possible therapy for the neurodegenerative phase of MS.

## Introduction

Multiple Sclerosis (MS) is an inflammation-mediated demyelinating disease of the central nervous system (CNS) and the leading cause of nontraumatic disability in young adults [1]. Classically, MS has been considered a demyelinating disease affecting white matter; however, there is growing evidence that axonal/neuronal damage and grey matter loss/neurodegeneration play a central role and contribute to sequelae [2–4].

The majority of available treatments for patients with MS are immune modulators because most research has been conducted using the experimental autoimmune encephalomyelitis (EAE) animal model. However, recent data have highlighted the importance of considering the neurodegenerative component of the disease that is reproduced in the model of Theiler’s murine encephalomyelitis virus (TMEV) [5–7]. TMEV infection in SJL/J mice causes chronic progressive demyelinating disease, with characteristics that resemble progressive forms of MS that entail brain atrophy [8]. Lesions induced in this animal model have both an inflammatory component and a neurodegenerative component, achieving a close resemblance to MS and therefore making the model suitable for analysing the effects of drugs that promote the repair of CNS lesions. In MS, inflammation and degeneration appear to occur simultaneously; a feature that is reflected in the TMEV model, which also reproduces the neurodegenerative component of the disease observed during the delayed phase in most patients and in progressive forms of the disease [9].

Recent data have shown that peripheral circulating cells produce factors that promote *in vivo* CNS myelination via extracellular vesicle (EV) signalling [10,11]. EVs are small, 40–120 nm membrane vesicles of endocytic origin produced by virtually all cells in the body, including mesenchymal stem cells (MSCs), which are capable of improving functional recovery, fibre tract integrity, axonal sprouting and white matter repair markers in neurological diseases [12–14].

Among the various types of stem cells used for the treatment of neurodegenerative diseases, MSCs appear to have aroused the greatest interest as promising candidates. Previous studies using adipose tissue-derived mesenchymal stem cells (ADMSCs) have shown their therapeutic potential in the EAE model by mediating immunomodulation and enhancing brain repair mechanisms [15]. Nevertheless, given that only a small proportion of injected stem cells can reach, engraft and differentiate at the lesion’s site suggests that the beneficial effect of stem cells can be indirect and depends on their paracrine activity [16]. Stem cells produce a wide spectrum of EVs, which contain cytokines, chemokines, growth factors, proteins and nucleic acids that might affect cell-to-cell communication. From a translational point of view, interest in EVs has increased in recent years, given they can easily be produced in large amounts and at lower cost than stem cells and can be stored for further use. Increasing evidence suggests that EVs display anti-inflammatory properties, reducing the number of activated inflammatory microglial cells, supporting oligodendrocytes and protecting neurons [11–14]. However, there is as yet no evidence regarding the therapeutic potential of EVs during the neurodegenerative phase of MS.

Here, we hypothesise that the administration of EVs derived from human ADMSCs might mediate repair mechanisms in CNS damage and promote recovery in TMEV-induced demyelinating disease (TMEV-IDD).

## Materials and methods

### Mesenchymal stem cell EV generation, isolation and characterisation

Human ADMSCs (Tebu-Bio, Spain) were thawed and suspended in conventional culture medium with Dulbecco’s Modified Eagle Medium (DMEM) (Gibco Laboratory, Grand Island, NY, USA) containing 20% fetal bovine serum (FBS; PAA Laboratories, GmbH, Pasching, Austria) and penicillin–streptomycin (Sigma-Aldrich, St Louis, MO, USA) in 75 cm^2^ tissue culture flasks (Nunc, Roskilde, Denmark). When the cells reached 90% confluence, the conventional culture medium was replaced with an EV-depleted FBS culture medium (System Biosciences, Mountain View, CA, USA). The cells were then cultured for an additional 24 hours, the media were collected and the EVs were isolated by ultracentrifugation. To discard the cells, the medium was centrifuged for 10 minutes at 300 x g. A second centrifugation for 10 minutes at 2000 x g was needed to discard the dead cells. Another centrifugation for 30 minutes at 10,000 x g was used to discard cell debris. To obtain EVs, the supernatant was centrifuged 70 minutes at 100,000 x g. Finally, we deleted the contaminating proteins of the EV pellet by centrifugation for 70 minutes at 100,000 x g and resuspended it in phosphate-buffered saline (PBS) (Sigma-Aldrich) [17].

For characterisation and further administration, the purified EVs were eluted in PBS and stored at -20°C until use. We have quantified the EVs by measuring the total protein concentration using a bicinchoninic acid commercial kit (Thermo Scientific, Waltham, MA, USA).

The EVs were characterised by studying morphology and size (<100 nm), using electron microscopy and NanoSight (NanoSight Ltd, UK); their phenotype expressing CD81 and CD63 markers was examined by western blot technique. The EV phenotype was determined by immunofluorescence using a specific lipophilic tracer, CellTrackerTM CM-DiI (Invitrogen, Oregon, USA), and was then colabelled with apoptosis-linked gene-2 interacting protein X (ALIX) (1:250, Abcam), a specific EV antibody.

### Animals and Theiler's virus infection

All animal care and experimental procedures were performed in accordance with EU (2010/63/EU) and governmental guidelines (Royal Decree 53/2013 BOE n°34 and Comunidad de Madrid: ES 280790000184) and were approved by the Ethics Committee on Animal Experimentation of the Cajal Institute (protocol number: 2013/03 CEEA-IC). All studies involving animals are reported in accordance with the Animal Research: Reporting of *in vivo* Experiments guidelines for reporting experiments involving animals [18]. A total of 33 animals were used in the experiments described here.

**Fig 1:**
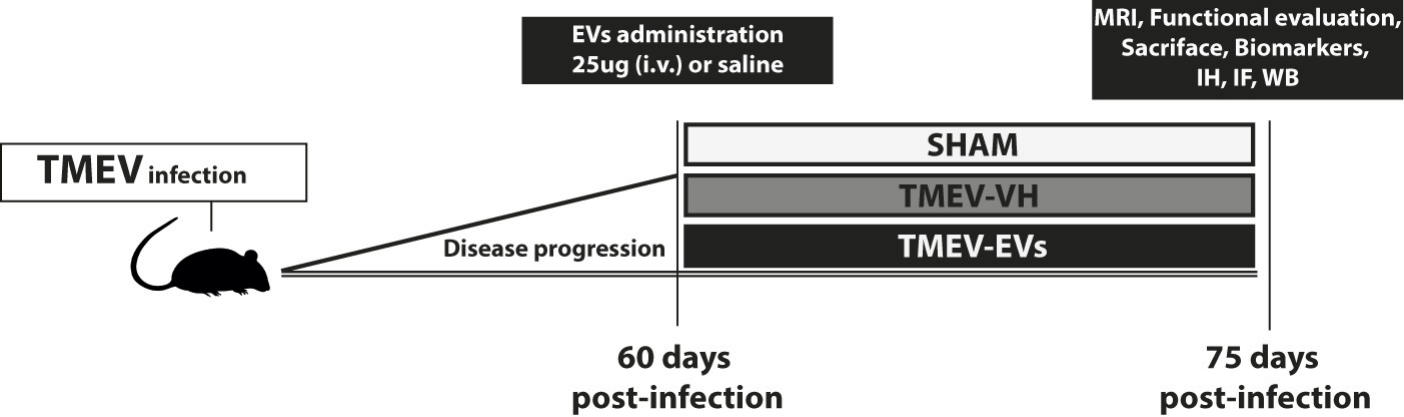
Experimental protocol scheme. EVs were isolated from human adipose tissue-derived MSCs and subsequently characterised. Four- to six-week-old female SJL/J mice were inoculated intracerebrally into the right hemisphere with 2 × 10^6^ plaque-forming units of the Daniel strain of TMEV. On day 60 postinfection, treatment (saline or EVs) was administered through the tail vein. Distribution of mice: sham (n = 7), TMEV-VH (n = 10) and TMEV-EVs (n = 10). On day 75 postinfection, we evaluated mouse behaviour and performed imaging, biomarker, histological and molecular studies. Abbreviations: TMEV: Theiler’s murine encephalomyelitis virus; EVs: extracellular vesicles; TMEV-EVs: Theiler’s murine encephalomyelitis virus with EV treatment; TMEV-VH: Theiler’s murine encephalomyelitis virus with saline treatment; MRI: magnetic resonance imaging; IH: immunohistochemistry; IF: immunofluorescence; WB: western blot.

TMEV-IDD-susceptible female SJL/J mice from Envigo (Barcelona, Spain) were maintained at our in-house colony (Cajal Institute, Madrid, Spain) on a 12-hour light/dark cycle with *ad libitum* access to food and water. Four- to six-week-old mice were intracranially inoculated in the right hemisphere with 2 × 10^6^ plaque-forming units of the Daniel strain of TMEV diluted in 30 μL of DMEM supplemented with 10% fetal calf serum (FCS) [19]. The sham-operated mice received 30 μL of DMEM + 10% FCS alone. On day 60 postinfection, 25 μg of EVs were administered to the treatment group (TMEV-EVs) through the tail vein. A saline injection of the same volume was delivered to the sham and nontreated TMEV (TMEV-VH) animals. Mice were distributed into the sham (n = 7), TMEV-VH (n = 10) and TMEV-EVs (n = 10) groups. The allocation of each animal to an experimental group was performed on the basis of a simple random choice: throwing a die (less than/equal to two: control; three or four: vehicle; and more than four: treatment). Details of the experimental protocol are shown in Fig 1.

### EV biodistribution

For the evaluation of EV biodistribution at 2 hours after intravenous administration, an additional six animals were used, which were divided into two groups: sham group (n = 3) and TMEV-EVs (n = 3). TAPA1 marker levels (CD81) (1:750, Abcam) were observed in the brain as well as in samples from the peripheral organs, such as the lungs, spleen and liver. A colabelling of EVs (CD63) with glial fibrillary acid protein (GFAP) (1:400, Millipore) and neuronal specific nuclear protein (NeuN) (1:100, Millipore) was made by immunofluorescence. The EV phenotype was determined by immunofluorescence with a specific lipophilic tracer, CellTrackerTM CM-DiI (Invitrogen, Carlsbad, CA, USA), and then colabelled with ALIX (1:250, Abcam). Images were acquired as a maximum confocal projection using a LEICA TCS SPE spectral confocal microscope (Leica Microsystems, Heidelberg, Germany) and analysed using LAS AF software (Leica). Mean fluorescence intensity was measured by the NIS-Element AR (Nikon) 4.5 Program.

### Behavioural analysis: Spontaneous motor, activity memory and object recognition test

Behavioural tests were evaluated on day 15 after treatment. For spontaneous locomotor activity, an activity cage coupled with a Digiscan analyser was used (Digiscan Activity Monitoring System, Omnitech Electronics Inc, Columbus, OH, USA). The number of times the animals broke the vertical sensor beams was automatically measured in two 5-minute sessions, as previously described [20]. Memory was evaluated using passive avoidance (Passive Avoidance Cage for Mice, Harvard Apparatus, MA, US), a fear-aggravated test in which mice learn to avoid a noninvasive foot shock. Briefly, the cage is composed of two compartments (light and dark). The test was conducted over 3 days: The animals were free to explore both compartments during the first day, preferring to stay on the dark side due to their rodent nature; on the second day, a noninvasive foot shock was delivered in the dark compartment; and on the third day, the latency to enter the dark compartment was measured.

### *In vivo* magnetic resonance imaging

Ventricular size was analysed at 15 days after treatment by MRI (Bruker Pharmascan, Ettlingen, Germany) [21], using 7 Tesla horizontal bore magnets and T2-weighted (T2-W) spin-echo anatomical images acquired with a rapid acquisition with relaxation enhancement (RARE) sequence in axial orientations, with the following parameters: number of echo images, 2 (TE: 29.54 ms and 88.61 ms); TR, 3000 ms; RARE factor, 4; Av, 3; FOV, 3.5 cm; acquisition matrix, 256 × 256 corresponding to an in-plane resolution of 137 × 137 μm^2^; slice thickness, 1.00 mm without gap; and number of slices, 16. For this procedure, the animals were anesthetised with a 2% isoflurane-oxygen mixture in an induction chamber and the flow of anaesthetic gas was constantly regulated to maintain a breathing rate of 50 +/− 20 bpm.

Ventricular size was calculated as a percentage of whole brain volume in single-slice regions of interest. For quantification, all the images were processed using the Image J 1.52D program (NIH software, Bethesda, MD, USA).

### Tissue processing, immunohistochemistry, immunofluorescence and western blot

The mice were anesthetised with pentobarbital (50 mg/kg body weight, intraperitoneally) and perfused with saline. The animals’ brains and spinal cords were fixed overnight in 4% paraformaldehyde prepared in 0.1 M phosphate buffer (PB), and cryoprotected in 30% sucrose solution in 0.1 M PB, then mounted in optimal cutting temperature compound (Sakura Finetek, CA, USA) and stored at -80°C. Coronal brain and spinal cord sections (30 μm thick) were obtained using a Leica CM1950 cryostat (Leica) and processed for the following studies.

For the immunohistochemistry, the inflammatory infiltrate studies were obtained by hematoxylin and eosin stain. Slides were immersed 1 minute in hematoxylin and 3 minutes in eosin previous to water washes. Finally, they were dehydrated and coverslipped with Depex. Infiltrates were observed in spinal cord sections, using a 20× objective lens, and were processed by image analysis software (Image-Pro Plus 4.1, Media Cybernetics). The number of lesions were evaluated on a scale of 0 to 4, the score reflecting the number of infiltrates in the thoracic spinal cord sections. A score of 4 reflects the largest number of infiltrates with all the intermediate scores (1, 2, and 3) to define the increase in the density of infiltrates in the spinal cord tissue.

In the cell proliferation studies, slides were treated with citrate buffer (pH 6) at 95°C for 10 minutes; after inhibiting endogenous peroxidase, they were blocked for 1 hour with 3% normal goat serum (Vector Laboratories) at room temperature. The slides were incubated overnight with primary polyclonal antibody anti-Ki67 (1:100; Chemicon, Temecula, CA, USA). After having been washed in Tris-buffered saline (TBS), they were incubated with biotinylated universal secondary antibody and in streptavidin/peroxidase (Vector Laboratories, Burlingame, CA, USA). The sections were again washed in TBS and incubated with chromogen 3,3-diaminobenzidine (DAB) (Invitrogen). Finally, they were dehydrated and coverslipped. Cell proliferation was analysed in the brain sections corresponding to the subventricular zone (SVZ) of each animal. The number of positive cells was counted, using a 40× objective lens, and was processed by image analysis software (Image-Pro Plus 4.1, Media Cybernetics).

For immunofluorescence of the brain, we used antibodies against 2',3'-cyclic-nucleotide 3'-phosphodiesterase (CNPase) (1:500, Sigma-Aldrich), GFAP (1:400, Millipore), Iba-1 (1:1000, Millipore) and myelin basic protein (MBP) (1:200, Abcam). In the spinal cord sections, we used CNPase (1:500, Sigma-Aldrich) and Iba-1 (1:1000, Millipore). Anti-mouse and anti-rabbit Alexa Fluor 488 (1:750, Invitrogen) were used as secondary antibodies. All sections were mounted with H-1200 VectaShield mounting medium for fluorescence with 4',6-diamidino-2-phenylindole (ATOM). To quantify the levels of both markers, mean fluorescence intensity was measured under the confocal microscope using a 40x objective lens in a LEICA TCS SPE spectral confocal microscope (Leica), and the confocal images were analysed using LEICA software LAS AF, version 2.0.1 Build 2043. Images were acquired as a maximum confocal projection and analysed using LAS AF software (Leica).

For western blot analysis, the same antibodies (CNPase [1:1000, Sigma-Aldrich], GFAP [1:500, Millipore], Iba-1 [1:1000, Millipore] and MBP [1:100, Abcam]) were used. Units were normalised based on ß-actin levels (1:400, Sigma-Aldrich). Normalised optical density was calculated using the Alliance imaging system (UVItec Ltd, Cambridge, UK) (n = 4 animals per group).

### Determination of cytokine plasma levels by multiplex ELISA assay

Plasma samples were analysed by multiplex enzyme-linked immunosorbent assay (Affymetrix, eBioscience). An array of 12 cytokines was analysed (Mouse ProcartaPlex Panel Th1/Th2 + IL17A): granulocyte-macrophage colony-stimulating factor (GM-CSF), interferon gamma (IFNγ), tumour necrosis factor alpha (TNFα) and interleukin (IL)-1β, IL-2, IL-4, IL-5, IL-6, IL-12p70, IL-13, IL-17A and IL-18, using a Luminex—LX200 Analyzer (Luminex Corporation, Austin, TX, USA) and MILLIPLEX Analyst 5.1 software. Assay Sensitivity: GM-CSF: 0.19 pg/mL; IFNγ: 0.09 pg/mL; TNFα: 0.39 pg/mL; IL-1β: 0.14 pg/mL; IL-2: 0.10 pg/mL; IL-4: 0.03 pg/mL; IL-5: 0.32 pg/mL; IL-6: 0.21 pg/mL; IL-12p70: 0.21 pg/mL; IL-13: 0.16 pg/mL; IL-17A: 0.016 pg/mL; IL-18: 9.95 pg/mL.

### Statistics

The data are expressed as mean ± standard deviation. A one-way analysis of variance (ANOVA) followed by the Bonferroni *post-hoc* test, or a Kruskal–Wallis ANOVA followed by the Mann–Whitney U test were used to determine statistical significance. Values of p < .05 were considered significant at a 95% confidence interval; the data were calculated using statistical software programs IBM SPSS statistics 22 and GraphPad 7.0.

## Results

### EV characterisation and biodistribution

**Fig 2:**
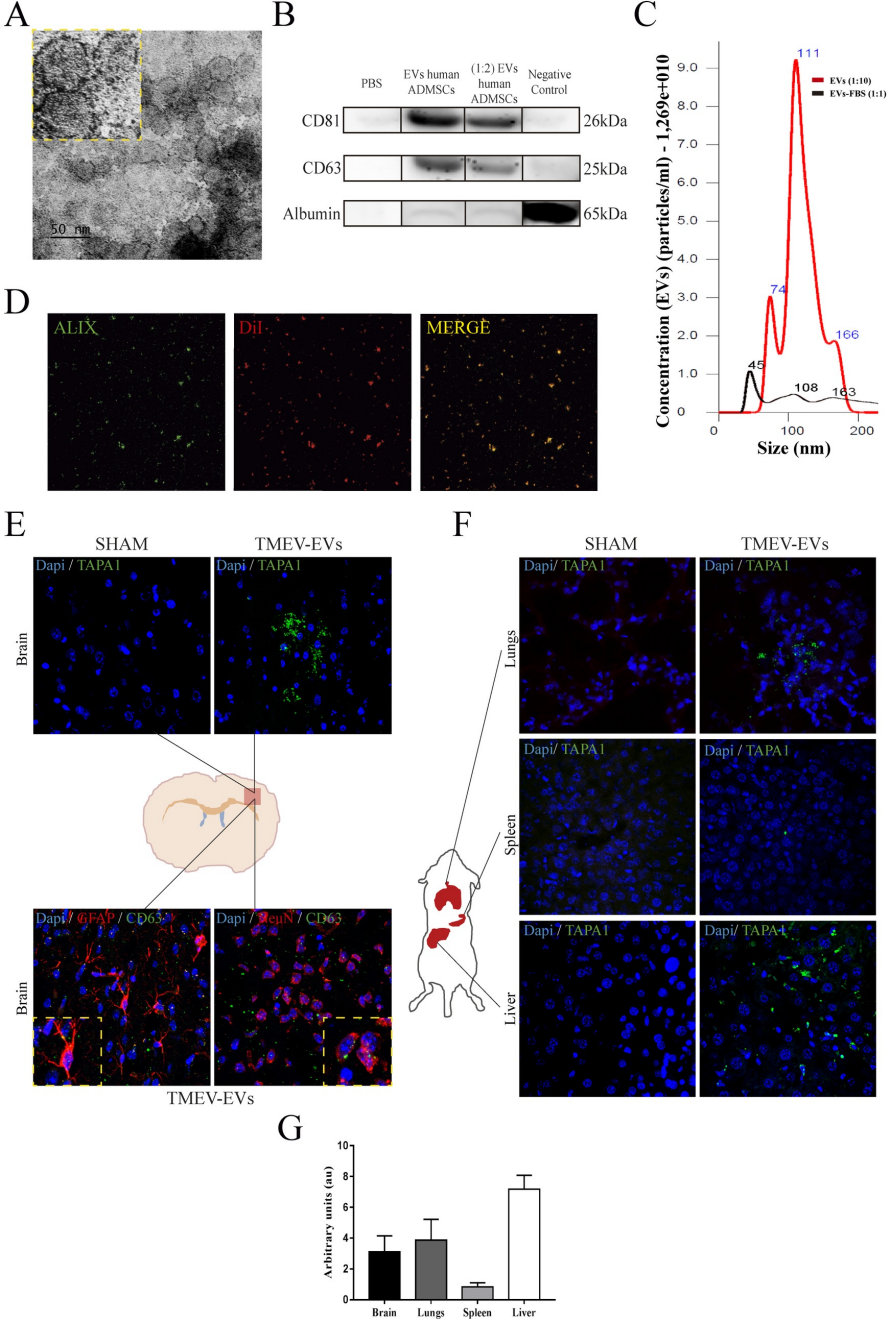
EV characterisation and biodistribution after intravenous administration. EVs were characterised using various techniques: **(A) Electron microscope image.** EVs with size smaller than 100 nm were observed by electron microscope. **(B) Western blot.** Detection of EVs with specific markers (positive: CD81 and CD63; negative: albumin) by western blot assay. Negative control samples are supernatant with debris and dead cells. The gels image was cropped. **(C) Characteristics of particles.** Size and concentration of the particles detected in the sample EVs isolated and EVs-FBS (culture media) by NanoSight. **(D) Phenotype of EVs.** Immunofluorescence of EVs labelled with DiI (red) and the specific EV marker antibody anti-ALIX (green). **(E) Biodistribution of the EVs in the brain.** Top: after intravenous administration, EVs were observed in the brain by the TAPA-1 marker (green), using immunofluorescence. Bottom: Colabelling with EVs (green) and cellular markers (GFAP/NeuN) (red) in the brain, using immunofluorescence. **(F) Biodistribution of the EVs in peripheral organs.** EVs were found in various peripheral organs, such as the lungs, spleen and liver, by immunofluorescence (green). **(G) EV quantification.** Fluorescence intensity quantification (arbitrary units) in brain and peripheral organs. Abbreviations: EVs: extracellular vesicles; human ADMSCs: human adipose tissue-derived mesenchymal stem cells; EVs-FBS: extracellular vesicles -depleted FBS media; DiI: lipophilic Tracer CellTrackerTM CM-DiI; GFAP: glial fibrillary acidic protein; NeuN: neuronal specific nuclear protein.

The EVs that we isolated came from a culture of 2x10^6^ human ADMSCs. Per flask, we obtained approximately 9 μg/ul of EVs. Regarding EV characterisation, EVs showed typical morphology and size (<100 nm) by electron microscope (Fig 2A). EVs were detected by specific markers (positive: CD81 and CD63; negative: albumin), using western blot assay (Fig 2B). We confirmed the size (<100 nm) with a 1,269e+010 particles/ml concentration using NanoSight (Fig 2C). Their phenotype was ALIX+ (EV-specific marker) according to immunofluorescence (Fig 2D). EV biodistribution in the TMEV-infected mice was assessed by TAPA1/CD81 staining as a positive marker in the brain (top of Fig 2E). EVs (CD63) were found in the brain (bottom of Fig 2E) as well as in the peripheral organs, such as the lungs, spleen and liver (Fig 2F), using colabelling with cellular markers such as GFAP and NeuN, 2 hours after their intravenous administration. Finally, we measured fluorescence intensity (arbitrary unit [A.U.]) in the brain (3.11 ± 1.03 A.U.) and peripheral organs such as the lungs (3.86 ± 1.36 A.U.), spleen (0.84 ± 0.26 A.U.) and liver (7.17 ± 0.91 A.U.).

### Effects of EV treatment on mouse behaviour, brain atrophy imaging, spinal cord inflammatory infiltrates and cell proliferation in the subventricular zone

**Fig 3:**
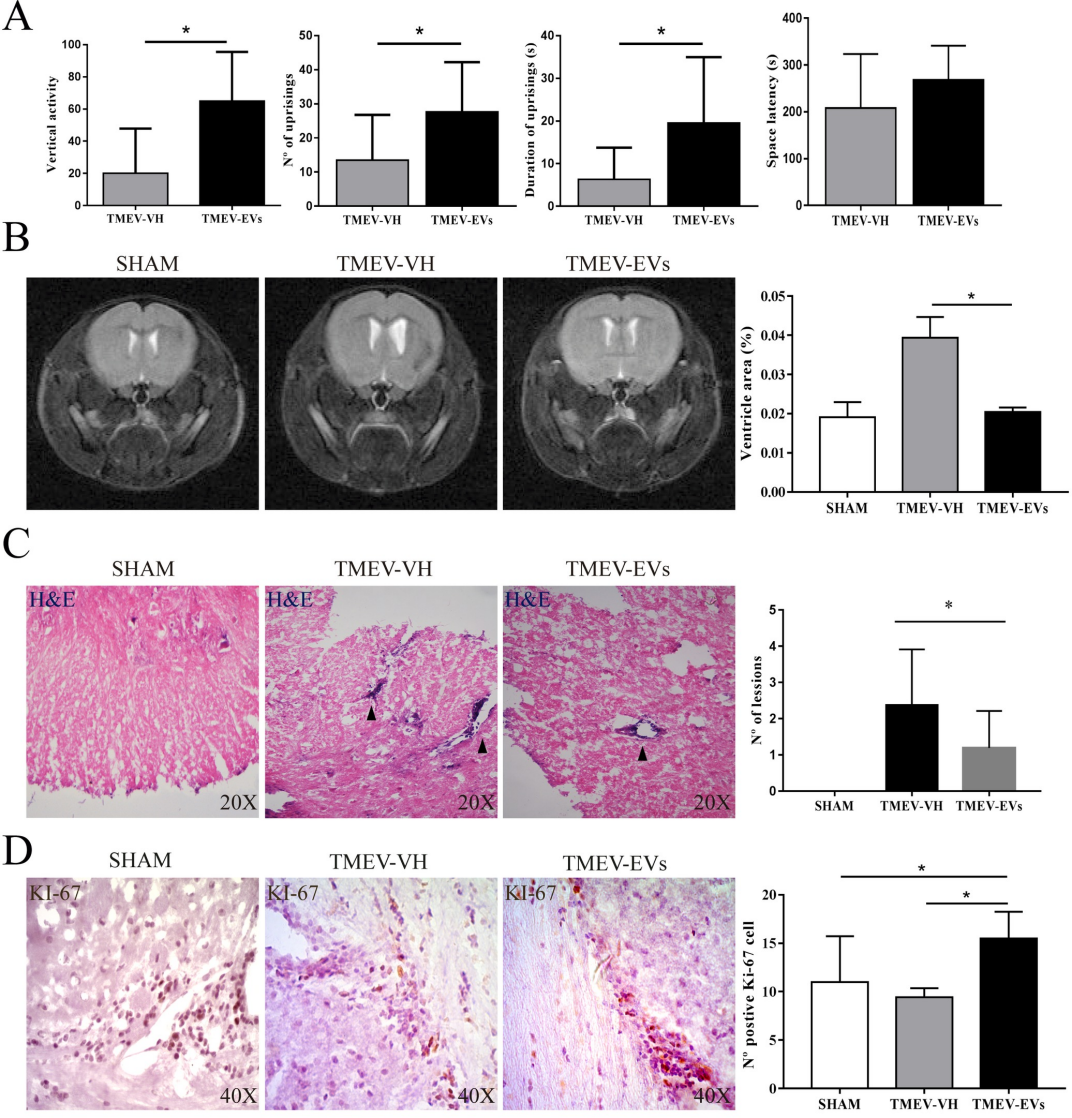
**(A) Behavioural evaluation.** Behaviour was evaluated by various tests: activity-cage, passive avoidance and object recognition (n = 7 animals in the sham group; n = 10 animals each in the TMEV-VH and TMEV-EVs groups). Data are mean ± SD, *p < .05. **(B) Ventricular size.** Qualitative and quantitative analysis of T2-weighted MRI images showed significant differences in ventricle sizes (coronal) between the TMEV-VH and TMEV-EVs groups (n = 4 animals per group). **(C) Hematoxylin and eosin stain.** Spinal cord sections, with hematoxylin and eosin stain, in which inflammatory infiltrates can be observed (black arrows) in the TMEV-VH and TMEV-EVs groups (n = 4 animals per group). **(D) Cell proliferation**. KI-67-positive cells in the SVZ (n = 10 animals per group). Data are mean ± SD, *p < .05. Abbreviations: EVs: extracellular vesicles; SVZ: subventricular zone; TMEV: Theiler’s murine encephalomyelitis virus; TMEV-EVs: Theiler’s murine encephalomyelitis virus with EV treatment; TMEV-VH: Theiler’s murine encephalomyelitis virus with saline treatment.

The TMEV-infected mice showed diminished vertical activity in the chronic phase of the disease, consistent with other studies [20,22]. Activity-cage testing showed that the TMEV mice treated with EVs (TMEV-EVs) displayed better motor activity than the TMEV-VH group; specifically, in the vertical activity (61.5 ± 7.88 *vs*. 21.3 ± 15.64, respectively; p < .05), number of times stood on hind legs (27.67 ± 4.85 *vs*. 13.44 ± 4.48, respectively; p < .05) and time spent standing on hind legs (19.56 ± 4.87 *vs*. 6.29 ± 2.36, respectively; p < .05). In the passive avoidance memory test, mice showed a trend toward better performance in the TMEV-EVs group (261 ± 79.31) compared with the TMEV-VH group (203.64 ± 114.87), without reaching statistical significance (Fig 3A). Therefore, intravenously administered EVs improved motor function in TMEV mice. We then evaluated the effect of EV treatment on imaging brain atrophy; thus, relative ventricular size in the coronal plane was evaluated for the sham, TMEV-VH and TMEV-EVs groups, taking into account the size of the ventricles with respect to the total brain size. The TMEV-EVs group showed that the ventricle size (0.020 ± 0.0011) was smaller than that of the TMEV-VH group (0.039 ± 0.005) and similar to that observed in the sham group (0.020 ± 0.0038) (Fig 3B).

In addition, we investigated the effect of EV treatment on spinal cord inflammatory infiltrates and cell proliferation in the SVZ niche. In both the TMEV-VH and TMEV-EVs groups, inflammatory infiltrates were located in the white matter of the spinal cord. The number of lesions was significantly higher in the TMEV-VH group (2.38 ± 1.532) compared with the TMEV-EVs group (1.19 ± 1.020; p < .05) (Fig 3C), suggesting that EV administration modulates inflammation in TMEV mice.

Proliferative KI-67^+^ cells were observed in the TMEV-VH mice, in agreement with previous studies showing increased cell proliferation in the SVZ in this model of MS [23,24]. The number of KI-67^+^ cells in the SVZ was significantly higher in the TMEV-EVs group (15.50 ± 2.759) compared with the TMEV-VH group (9.40 ± 0.966; p < .05) and with the sham group (11 ± 4.726; p < .05), as shown in Fig 3D, indicating that intravenously administered EVs promote cell proliferation in the SVZ.

### Effects of EV treatment on glial marker and myelin protein expression in the brain and spinal cord of TMEV mice

**Fig 4:**
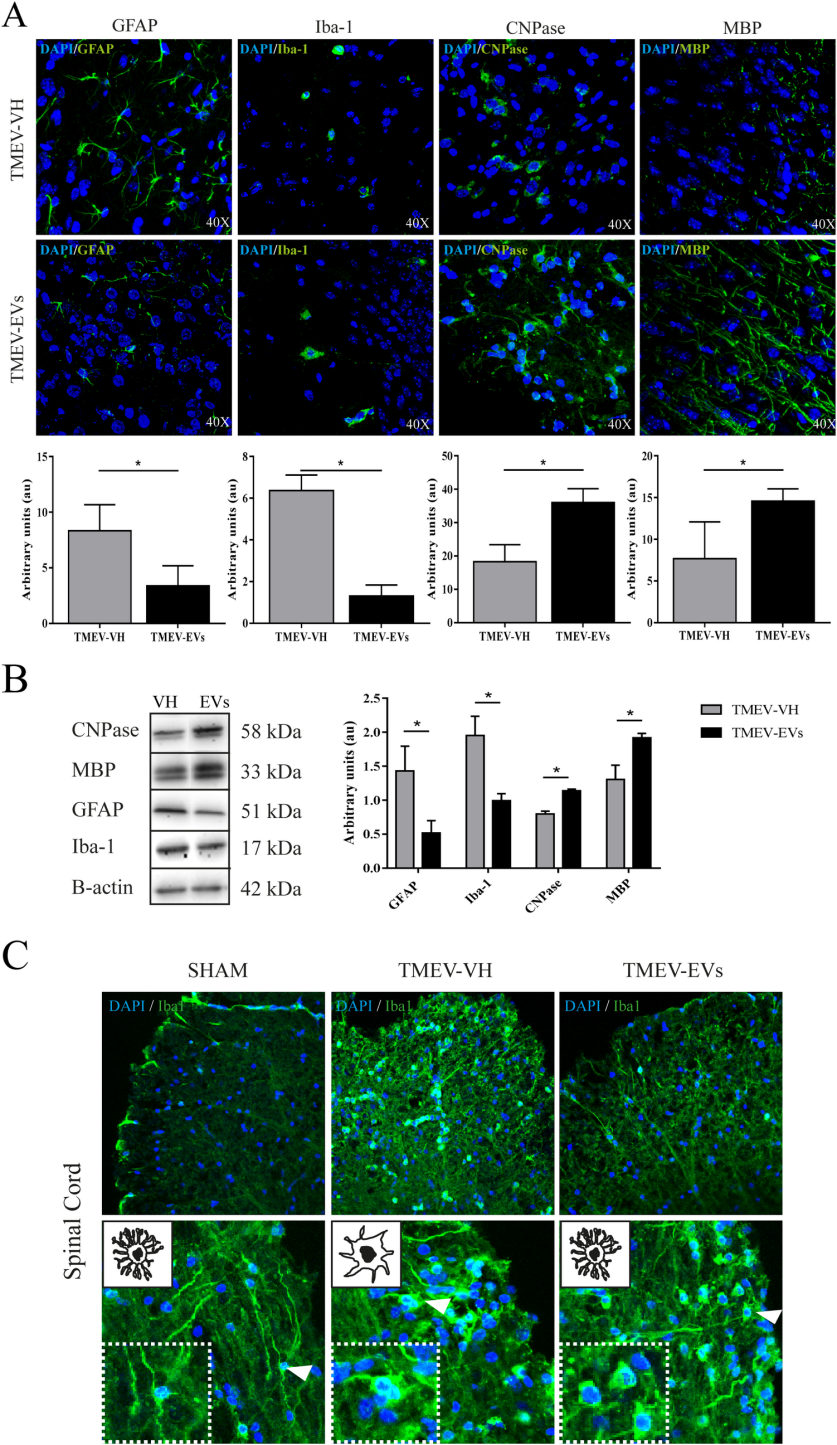
Brain repair-associated markers after EV treatment at 2 weeks post-treatment. **(A) Immunofluorescence of brain repair markers.** Significant difference in brain repair marker expression (GFAP, Iba-1, CNPase and MBP) (green) between the TMEV-VH and TMEV-EVs groups by immunofluorescence (n = 4 animals per group). Data are mean ± SD, *p < .05. **(B) Western blot.** Brain repair marker expression (GFAP, Iba-1, CNPase and MBP) with their quantifications by western blot. Data are mean ± SD, *p < .05. **(C) Immunofluorescence in spinal cord.** Morphological changes in microglia activation in spinal cord by immunofluorescence with Iba-1 (20X, 40X) (green). EVs: extracellular vesicles; VH: vehicle; TMEV-EVs: Theiler’s murine encephalomyelitis virus with EV treatment; TMEV-VH: Theiler’s murine encephalomyelitis virus with saline treatment; CNPase: 2',3'-cyclic-nucleotide 3'-phosphodiesterase; MBP: myelin basic protein; GFAP: glial fibrillary acidic protein.

Brain immunofluorescence analysis at day 15 post-EV treatment (Fig 4A) showed that the group of mice that intravenously received EVs exhibited a significant reduction in the fluorescence intensity of glial markers—GFAP for astrocytes (3.37 ± 1.80 A.U.) and Iba-1 for microglia (1.30 ± 0.53 A.U.)—compared with the TMEV-VH group (8.34 ± 2.34 vs. 6.36 ± 0.75 A.U., respectively; p < .05). In addition, we observed a significant increase in myelin proteins such as CNPase (36.02 ± 4.15 vs. 18.24 ± 5.14 A.U., respectively; p < .05) and MBP (14.57 ± 1.48 vs. 7.67 ± 4.43 A.U., respectively; p < .05) in the TMEV-EVs group compared with the TMEV-VH mice (Fig 4A). The immunofluorescence data were confirmed by western blot analysis because we found a significant reduction in GFAP in the brains of the TMEV-EVs mice (0.52 ± 0.18 A.U.) compared with the TMEV-VH group (1.43 ± 0.36 A.U.; p = .009). Regarding Iba-1 expression, the TMEV-EVs group also showed lower values (0.99 ± 0.10 A.U.) compared with the TMEV-VH group (1.95 ± 0.28 A.U.; p = .004). Moreover, when comparing the TMEV-EVs and TMEV-VH groups, there were higher levels of CNPase (1.14 ± 0.23 vs. 0.795 ± 0.04 A.U., respectively; p = .05) as well as of MBP (1.92 ± 0.06 vs. 1.30 ± 0.21 A.U., respectively; p = .007) (Fig 4B). With respect to the spinal cord, we did not find significant differences between the TMEV-EVs and TMEV-VH groups in any of the above markers by western blot: GFAP (0.5 ± 0.06 vs. 0.49 ± 0.14 A.U., respectively); Iba-1 (0.65 ± 0.04 vs. 0.67 ± 0.04 A.U., respectively); CNPase (0.51 ± 0.05 vs. 0.59 ± 0.09 A.U., respectively); and MBP (0.71 ± 0.11 vs. 0.42 ± 0.23 A.U., respectively); or by immunofluorescence (data not shown). Nevertheless, we noticed some changes in the morphology of the microglial cells (Fig 4B), from cells with ramified processes in sections from the sham mice to more rounded cells with short processes in sections from the TMEV mice. This was clearly indicative of spinal cord neuroinflammation in the TMEV-VH mice, in agreement with numerous other reports [25,26]. In the TMEV mice treated with EVs, we found that the morphology of the microglial cells was similar to that observed in the sham mice, suggesting that EV administration modulates inflammation in the spinal cord of TMEV mice.

### EV treatment reduces plasma cytokine levels in the TMEV model in the chronic progressive phase

**Fig 5:**
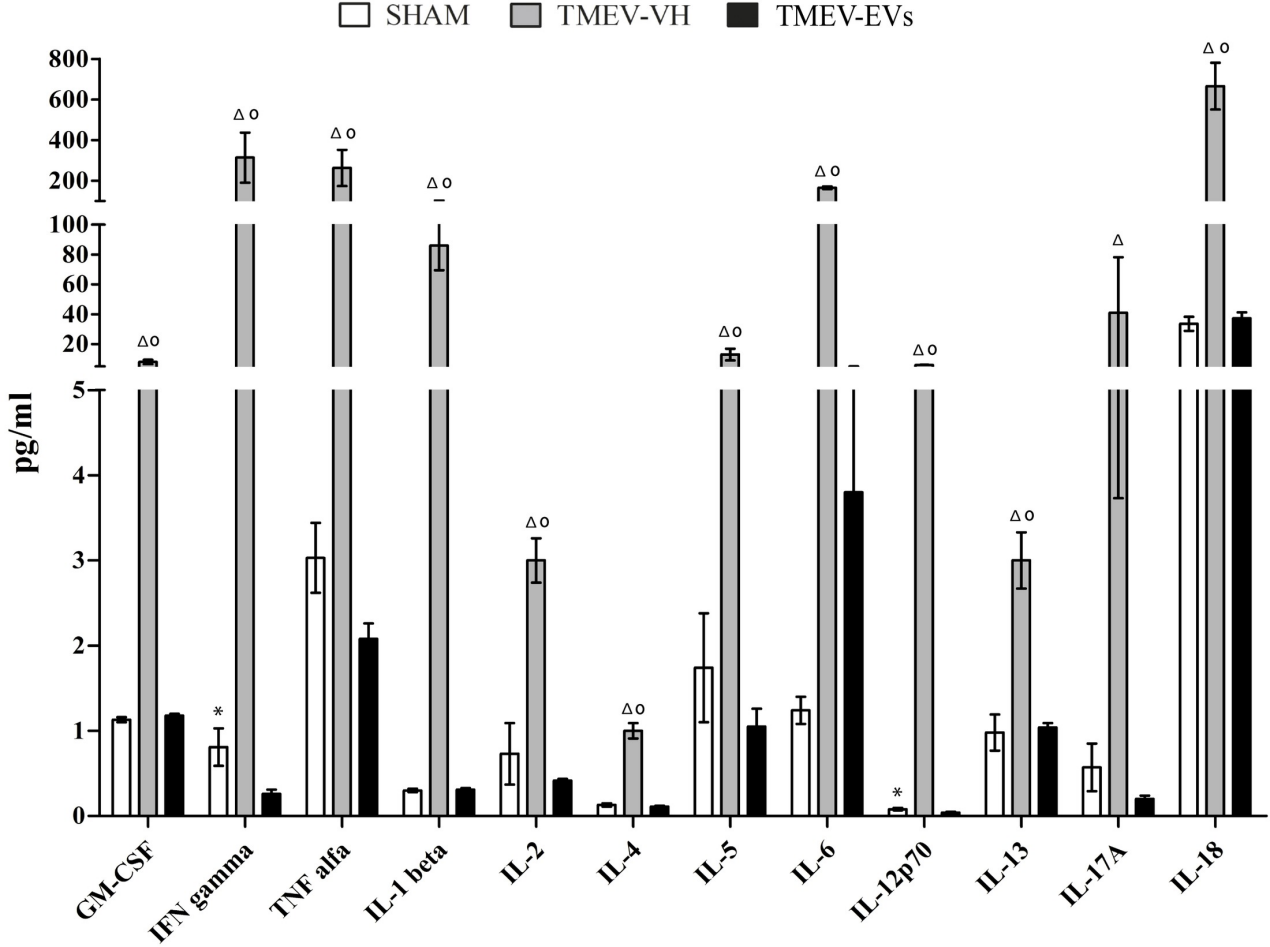
Plasma levels of cytokines analysed at the chronic phase and 2 weeks after EV treatment. The TMEV-VH group showed higher levels of these cytokines in plasma compared with the treated group (n = 7 animals in the sham group; n = 7 animals in the TMEV-VH group and n = 12 animals in the TMEV-EVs group). Data are mean ± SEM, *p < .05 sham *vs*. TMEV-EVs; ^Δ^p < .05 TMEV-VH vs. TMEV-EVs; ^о^p < .05 TMEV-VH vs. sham. Abbreviations: EVs: extracellular vesicles; TMEV-EVs: Theiler’s murine encephalomyelitis virus with EV treatment; TMEV-VH: Theiler’s murine encephalomyelitis virus with saline treatment.

To determine whether the modulation of inflammation observed in the CNS after EV administration also occurred at the systemic level in the chronic progressive phase of the disease, we analysed the levels of 12 Th1/Th2 cytokines as well as IL17A in plasma obtained from the three groups of mice. As shown Fig 5, there were significantly higher levels of all the cytokines tested in the TMEV-VH group compared with the TMEV-EVs group: GM-CSF (8.98 ± 1.44 vs. 1.18 ± 0.02 pg/mL, respectively), IFNγ (314.91 ± 123.16 vs. 0.26 ± 0.05 pg/mL, respectively), TNFα (263.34 ± 89.01 vs. 2.08 ± 0.18 pg/mL, respectively), IL-1β (86.63 ± 16.49 vs. 0.308 ± 0.02 pg/mL, respectively), IL-2 (3.26 ± 0.26 vs. 0.416 ± 0.02 pg/mL, respectively), IL-4 n.d.; IL-5 (13.258 ± 3.92 vs. 1.05 ± 0.21 pg/mL, respectively), IL-6 (166.14 ± 6.47 vs. 3.80 ± 1.33 pg/mL, respectively), IL-12p70 (6.54 ± 0.09 vs. 0.04 ± 0.01 pg/mL, respectively), IL-13 (3.88 ± 0.33 vs. 1.04 ± 0.05 pg/mL, respectively), IL-17A (41.83 ± 37.27 vs. 0.2 ± 0.04 pg/mL, respectively) and IL-18 (666.14 ± 115.26 vs. 23 ± 4.05 pg/mL, respectively) (p<.05). As expected, we found significant differences between the control sham mice and the TMEV-VH mice; the TMEV-VH mice showing increased plasma levels of all the GM-CSF, IFNγ, TNFα, IL-1β, IL-2, IL-4, IL-5, IL-6, IL-12p70, IL-13 and IL-18 cytokines. In addition, we detected a significant difference (p˂.05) in plasma levels of IFNγ and IL-12p70 between the sham and TMEV-EVs groups, showing reduced levels of both cytokines in the TMEV-EVs mice (Fig 5). Interestingly, both, pro- and anti-inflammatory cytokines, such as IL-5 and IL-13, were downregulated following EV treatment, although to a different degree. The most relevant results correspond to IFNγ, TNFα and IL-1βtogether with IL-18 of the IL-1 family, IL-6 and IL-12 p70 the bridge between innate and acquired immunity, and IL-17 A, one of the main pathogenic cytokines in MS.

## Discussion

The results of this study showed that intravenous EV administration was able to improve motor deficits in the TMEV-IDD model, an experimental model of primary progressive MS. We found that the treated group had better functional recovery than the mice receiving vehicle. The group treated with EVs from ADMSCs also had higher levels of white matter-associated markers (CNPase and MBP) than the control group, suggesting that EV administration might induce repair of white matter fibre tracts.

In this model, central atrophy accompanies the demyelinating condition [27]. Here, we determined the rate of brain atrophy and its relationship to disability by the activity cage assay. Our results demonstrated the development of significant brain atrophy in parallel with impaired vertical activity. ADMSC administration is considered to be an appropriate therapeutic strategy because ADMSCs enhance the natural repair processes of the brain after injury [28,29]. However, the mechanisms underlying these repair processes are still unknown. A wealth of findings have shown the role of EVs in nerve regeneration, neuronal protection and remyelination, indicating the possible therapeutic potential of these vesicles in neurological diseases [11,13,14,30,31]. In our study, we have demonstrated that the intravenous administration of EVs from ADMSCs exerts therapeutic effects on the TMEV model, improving motor disability and reducing brain atrophy. These beneficial effects were accompanied by a decreased in the number of inflammatory infiltrates in the spinal cord and increased cell proliferation in the SVZ, although at this time we do not comprehend the meaning of this increase regarding putative repair mechanisms; further studies are being performed to shed light on this issue. We report here a possible mechanism previously associated with ADMSC EV therapy function, such as the modulation of neuroinflammation, consistent with many other studies [31–33]. In this regard, EV administration reduced several glial markers—such as GFAP for astrocytes and Iba-1 for microglia in the brain—as well as the morphologic characteristics of microglial cells towards a less inflammatory phenotype within the spinal cord of TMEV mice. Remarkably, the drastic reduction in plasma cytokine levels, mainly of the Th1 and Th17 phenotypes in TMEV mice treated with EVs, constitutes clear evidence for the immunomodulatory ability of intravenous EVs. Cumulative evidence has indicated that systemic cytokines might act as mediators of CNS damage, including demyelination, via neuroinflammatory pathways [34,35]. In addition, cytokines in the blood can alter peripheral immune cells and promote interactions with the blood brain barrier (BBB), affecting peripheral monocytes and T and B lymphocytes, inhibiting their entry into the CNS. There are numerous examples that show how a modulatory effect on systemic immunity can affect the symptomatology in experimental models of MS [36]. Among others, the anti-inflammatory activity of EVs from ADMSCs might underlie their benefits in our progressive MS model. Nevertheless, at present we cannot determine the extent to which the downregulation of plasma cytokines impacts on TMEV progression or the specific cellular target of their actions. We are unable to establish unequivocally whether the beneficial effects we observed in the progressive MS model are mediated by systemic immune modulation or by subsequent actions on neural cells. Further studies are needed to identify the exact mechanisms of EV-induced improvement of TMEV-IDD progression.

There remains an incomplete picture regarding the role of EVs in MS pathogenic mechanisms, their importance as biomarkers and their potential as therapeutic targets. With respect to therapeutic applications, understanding the *in vivo* biodistribution after systemic delivery is of great relevance [37]. Despite intense research on the subject, few studies have analysed EV biodistribution. Using fluorescently-labelled EVs, we observed an accumulation of green fluorescence not only in various peripheral organs such as the lung, spleen and liver but also in the brains of the TMEV mice at 2 hours after intravenous administration as a consequence of the capacity to pass through the BBB. Our observations suggest that EVs could be one of the factors involved in the communication between the CNS and the peripheral immune system, mediating the modulation of both central and systemic responses following brain TMEV infection, as has been considered recently in other models of peripheral inflammation [32]. Due to the capacity of EVs to cross the BBB, they can be considered as therapeutic agents in many clinical studies as a delivery system to transport RNA, protein and drug molecules. The development of cell-free vesicle-based therapeutics is a particularly exciting application, because they can recapitulate the modulatory and regenerative potential of cell-based therapies without the intrinsic safety concerns connected with the administration of live cells. Regarding clinical applications, there is as yet no gold standard method for high production of EVs for patient treatment. However, the manufacture of the medicinal product should be performed by companies specialised for this purpose. Once the EV treatment is prepared, it could be stored at −80°C for at least 90 days at the hospital until needed; however, storage at below −70°C is the most favourable condition for long-term preservation of fresh EVs for clinical application and basic research [38]. EVs are considered an important diagnostic tool, given they can be detected in biological fluids, they are very stable and their contents are protected [39]. In this sense, vesicles have attracted great interest for their use as biomarkers, which has led to the development of EV-based commercial diagnostic kits [40].

### Conclusions

The present study is the first to demonstrate that intravenous administration of EVs derived from human ADMSCs attenuates motor deficits through immunomodulatory actions, diminishing brain atrophy and promoting remyelination in a murine model of progressive MS. Nevertheless, further studies are necessary to understand the complex molecular mechanisms that might influence the efficacy of EV delivery as a possible therapy for the neurodegenerative phase of MS.
